# The discriminatory politics of knowledge production

**DOI:** 10.1186/s12992-025-01173-w

**Published:** 2025-12-23

**Authors:** Fareeda Abo-Rass, Jesse B. Bump

**Affiliations:** 1 Takemi Program in International Health, Harvard T.H. Chan School of Public Health, 665 Huntington Avenue, Building 1, Room 1210, Boston, MA 02115 USA; 2 Department of Global Health and Population, Harvard T.H. Chan School of Public Health, Boston, MA USA

**Keywords:** Academic publishing, Peer review, Editorial gatekeeping, Knowledge production, Decolonial perspectives

## Abstract

Academic publishing is one of several forces that shape what is recognized as global health knowledge. The peer review process is meant to ensure rigor and quality, yet it can reproduce political and structural inequalities, especially when research challenges dominant narratives. For researchers from marginalized and colonized communities, these dynamics determine whether their language, identity, and lived realities are permitted in scholarly spaces. When political, historical, and socio-legal context is minimized or replaced with state-sanctioned labels, the result is not neutrality but the silencing of essential truths that directly shape health and mental health. This Comment examines how editorial and peer review practices operate as gatekeeping mechanisms that privilege dominant geopolitical narratives and marginalize Indigenous and decolonial perspectives. Drawing on a recent case where a peer-reviewed article, recommended for publication, faced subsequent editorial demands to replace politically accurate terminology referring to Palestinians, we show how language policing functions as epistemic control. These are not isolated incidents: global publishing norms pressure scholars toward state-sanctioned labels and “neutral” frames, sidelining colonial and political determinants of health. In global health, that pressure produces an evidence base that overlooks the sociopolitical conditions; occupation, systemic violence, legal segregation, displacement, that shape exposure, access, care pathways, and outcomes, including mental health. It produces an appearance of neutrality that is methodologically incomplete and ethically fragile, with downstream consequences for research agendas, funding priorities, program design, and accountability. Confronting the politics of knowledge production in global health requires structural change, not just diversity statements. Safeguarding researchers’ right to represent their communities in their own terms and embedding sociopolitical realities into analysis are essential. Without these changes, global health will continue to reproduce the inequalities it seeks to reduce, failing to generate knowledge that is genuinely global, representative, and just.

The publication peer-review system is a gatekeeping mechanism to maintain academic standards and ensure the integrity and quality of published knowledge. However, this gatekeeping can undermine these critical objectives in two complementary ways. Within academic rules and standards, reviewers may be biased against research that does not conform to mainstream approaches, for example, by applying known methods in novel ways, using interdisciplinary approaches, or investigating issues that are minoritized in some way. Perhaps the most widely known illustration of this bias is faced by qualitative researchers publishing in fields dominated by quantitative methods [1, 2]. The second form of bias occurs when the peer review process enforces dominant ideological and hegemonic narratives. In such cases, reviewers may dismiss research because it challenges existing power structures, mainstream perspectives, or political ideologies, even when the research is methodologically sound and well-designed. Particularly in the social sciences, this second form of bias disproportionately affects research involving Black, Indigenous, and People of Color (BIPOC) populations, including Palestinians [3]. Although this issue has been critically examined by Indigenous Palestinians in their own scholarly community [4], the obstacles they face, such as editorial efforts to erase Palestinian identity by enforcing ‘depoliticized terminology’ or framing requirements that obscure colonial realities, have yet to be publicized more generally. To bring visibility to this issue, we share our recent experience.

We encountered bias seeking to reinforce political ideology when we submitted an article to a journal published by a Swiss publisher last year. This incident occurred after the peer review had been completed and the reviewers had approved the manuscript for publication, and an associate editor became involved. Our study concerned an Arabic-language mental health literacy tool, designed to facilitate mental health research on Arabic-speaking populations in any setting. As part of the study, we administered the tool to three samples of Arabic-speaking participants, who happened to be Palestinian citizens of Israel. We mentioned this citizenship detail in the methods section in the interest of specificity, but our sample participants were chosen as speakers of Modern Standard Arabic, which is universally understood across the diverse accents and subcultures among Arabs in the Middle East and beyond.

The peer-review process lasted four months and involved four reviewers. Two were recommended for acceptance with minor revisions, while two requested major revisions. Notably, one of the latter described the paper as clear and well-structured and still recommended it. After submitting their reviews, both reviewers who had requested major revisions later withdrew from the process for unspecified reasons. Nonetheless, we took all feedback seriously and revised the manuscript accordingly.

Following this process, we were pleased to learn that the reviewers had recommended the manuscript for publication. However, in the very same letter, the associate editor raised concerns regarding terminology. He requested that we replace “Palestinian citizens of Israel” with “Israeli Arabs” or “Arabic-speaking Israelis”, arguing that these were legal terms—labels commonly used in Israeli state discourse. He also insisted on adding “in Israel” to the article’s title and claimed that our use of the term “Arab world” was inappropriate for a scientific article, deeming it political. In practice, the editor’s demands reflect a broader global pattern in which colonial powers, including within academic contexts, work to impose terminologies that serve their interests by regulating language in ways that suppress expressions of identity, culture, and political recognition, thereby erasing indigenous narratives [4]. This process, in turn, produces a discriminatory global health knowledge base that misrepresents reality and rests on partial rather than comprehensive evidence. The State of Israel exemplifies this dynamic. By insisting on terms such as “Israeli Arabs,” or using labels like “the Arab minority,” “Arabs in Israel,” “the Arab sector,” or even “the non-Jewish population,” it attempts to deny the historical and national identity of Palestinians. These state-sanctioned terms work to depoliticize Palestinians’ presence and obscure the legal, cultural, and territorial questions raised by their identity and history. However, for Palestinians, terms such as “Palestinian citizens of Israel” are not political provocations but essential expressions of identity, history, collective existence, culture, and justice; all of which are perceived by the state of Israel as forms of resistance.

We responded by explaining that our choice of terminology was intentional and aligned with established mental health research [5]. Regarding the title, we clarified that the study did not focus on Israel or its population per se; the inclusion of Palestinian citizens of Israel was simply part of the validation process requiring native speakers of Arabic. We maintained our position while thanking him for his feedback.

In response, the associate editor sent a lengthy email, accusing us of failing to adequately address reviewer concerns, even though the reviewers had already approved the manuscript for publication and were no longer involved in the review process. He criticized the study’s quality and ultimately stated that he would be forced to reject the article unless we complied with his demands. Faced with these evidently discriminatory editorial interventions, we withdrew our submission.

This experience is a clear example of editorial interference rooted in political considerations. It shows the role editorial authority can play in upholding dominant geopolitical narratives while sidelining alternative perspectives. The objection to the term “Palestinians”, paired with the insistence on using “in Israel”, demonstrates editorial alignment with state language, framed as a matter of procedural or legal correctness.

Palestinian researchers face bias regarding terminology and are also frequently and unfairly perceived as lacking scholarly objectivity, as demonstrated in Abu-Saad’s case [6]. Abu-Saad, a Palestinian professor at Ben-Gurion University, published an article in *American Behavioral Scientist* in 2008 titled *Where Inquiry Ends: The Peer Review Process and Indigenous Standpoints.* He detailed his attempt to publish a manuscript analyzing how the Israeli education system systematically de-educates Palestinians. Despite two of three peer reviewers recommending acceptance with revisions, one calling for further theoretical development, and another suggesting minor revisions, the editors rejected the submission. They justified their decision by stating that Abu-Saad approached the subject from a “particular point of view,” implying that his indigenous Palestinian identity was inherently biased.

As Abu-Saad quickly observed, the resistance he encountered was predicated on the assumption that dominant academic perspectives are neutral while indigenous perspectives are subjective. He pointed out that the reviewers, selected from the dominant majority, also had particular viewpoints that shaped their assessment. Yet, their perspectives were treated as objective and authoritative, while his was dismissed as inherently biased. Despite his attempts to engage in dialogue and revise the manuscript accordingly, the editors upheld their rejection, ultimately reinforcing the very academic suppression his study critiqued.

The review process Abu-Saad endured illustrates how the peer review system can be manipulated to silence marginalized narratives under the guise of academic objectivity. A similar dynamic was evident in our own experience, as the associate editor who had initially viewed the manuscript favorably shifted his focus to questioning the quality of our study. This raises concerns about how many studies by and about Indigenous populations may have been unjustly rejected on similar grounds, ultimately limiting the visibility and advancement of marginalized voices in academic discourse.

This pattern is not an isolated case, but part of a broader experience shared by Palestinian researchers. When we shared our case in a WhatsApp group of Palestinian researchers in Israeli academia, they immediately related. Many described how they had learned, often out of necessity rather than choice, to adopt the “accepted” language in their master’s theses and doctoral dissertations to avoid rejection. In Israeli universities, graduate Palestinian students are frequently advised to use official labels when discussing their own people. Consequently, researchers often avoid using the term “Palestinian” altogether, not because the approved official terms also reflect their identity, but only to conform to the unyielding expectations of an academic establishment shaped by the state’s political narrative.

Hegemonic ideology in Israeli academia influences terminology and also shapes broader conceptual frameworks and interpretations imposed by academic supervisors and institutional norms. Many Palestinian students are encouraged to frame their studies in ways that obscure the colonial and political structures that shape their reality. This practice systematically conditions young scholars to conduct research through imposed external lenses, detaching their work from the lived experiences of Palestinian communities and situating them instead within Israeli or Western epistemological frameworks.

Although these examples come from a specific local context, the underlying dynamics are widespread and cross-contextual. Similar patterns of exclusion confront Indigenous and marginalized scholars worldwide whenever their work challenges dominant academic norms. Boyd [3] notes how Indigenous researchers often internalize Western academic paradigms due to the systematic exclusion of Indigenous knowledge. The peer-review process disproportionately rejects Indigenous research, often because non-Indigenous scholars lack the expertise to evaluate it properly. Even without overt intention, reviewers can in this way pressure Indigenous scholars to modify their framing, language, and methodologies to fit dominant academic expectations.

## Structural barriers and the demand for fair and accurate knowledge production

The barriers Palestinian researchers face extend far beyond the examples noted above; across the global publishing landscape, they confront similar obstacles. Although they are held to the same expectations for frequent publication as their peers, they often encounter journals that act as gatekeepers, screening out their work rather than serving as platforms for genuine research. Such gatekeeping not only limits their opportunities to publish but also dictates how their research is framed, systematically divorcing it from its sociopolitical context. These methodological and ideological forms of exclusion are deeply intertwined, as the demand for apolitical framing often limits the kinds of methods and interpretations that are deemed acceptable. Meaningful scholarship on Palestinian communities is impossible without acknowledging the ongoing realities of colonialism, occupation, and systemic violence; realities thoroughly documented by scholars and human-rights organizations to include systemic dispossession, movement restrictions, legal segregation, and military occupation that shape nearly every aspect of daily life [7–10]. Despite these realities, studies on Palestinian education, mental health, and economic conditions are often conducted as if these aspects existed in isolation. Researchers are trained to prioritize quantifiable variables over critical discourse, resulting in publications that contribute to theoretical debates but fail to challenge or transform the oppressive structures they examine.

Pappé et al. [4] argue that the suppression of Palestinian perspectives is not merely exclusionary but an active effort to control and sanitize knowledge production by removing the colonial context that defines Palestinian existence. Academic institutions systematically depoliticize Palestinian studies, filtering them through frameworks of modernization, democratization, and cultural integration—an approach that we believe is particularly evident and especially pronounced in the case of Palestinian researchers in Israel. The barriers Palestinian scholars face in publishing their work are enforced through individual biases and part of a long-standing systemic effort to maintain Israeli and Western intellectual hegemony over Palestinian narratives. These challenges extend far beyond Palestinian scholars. Marginalized researchers worldwide, particularly those whose work challenges dominant narratives, frequently encounter similar barriers. Whether addressing colonial histories, racial inequalities, or social injustices, their scholarship is often scrutinized not for scientific rigor, but for the identity of the researchers.

Such exclusionary practices are embedded within the peer-review process itself. Review boards, editorial decisions, and disciplinary conventions systematically disadvantage Indigenous and marginalized perspectives, reinforcing existing power hierarchies. This is not a minor issue; it is a structural failure that perpetuates epistemic erasure and constrains the production of meaningful knowledge. Addressing these issues demands more than performative commitments to diversity. Academia must implement concrete changes, such as establishing an appeals process to challenge biased editorial decisions, creating a repository of experiences where marginalized researchers can document discrimination, and actively diversifying editorial boards to include scholars with decolonial perspectives. These reforms are essential to dismantling the exclusionary practices that have persisted for decades, practices that continue to silence the voices of Black, Native American, Latinx, LGBTQ+, and other marginalized scholars, as well as those who may belong to the mainstream demographically but whose perspective challenge academic norms. Equally essential is protecting the ability of researchers to define and represent their own communities authentically, while ensuring that scholarly analyses remain accurately grounded in sociopolitical realities. Without such critical reform, academia will continue to serve as a policing mechanism to uphold systemic inequalities, betraying its stated mission of advancing inquiry, knowledge, and fairness.

## Data Availability

No datasets were generated or analyzed during the current study.

## Abbreviations

BIPOC: Black, Indigenous, and People of Color
LGBTQ+: Lesbian, gay, bisexual, transgender, queer (or questioning), plus
UN OCHA: United Nations Office for the Coordination of Humanitarian Affairs
